# The effectiveness of virtual passport, an app-based intervention, for dementia care

**DOI:** 10.3389/fpsyt.2024.1457923

**Published:** 2024-09-26

**Authors:** Qian-Xi Hong, Wen-Fu Wang, Yuan-Han Yang, Yu-Chun Tung, Hong-Jie Dai, Wen-Chuin Hsu, Ling-Chun Huang, Kai-Ming Jhang

**Affiliations:** ^1^ Department of Neurology, Changhua Christian Hospital, Changhua, Taiwan; ^2^ Department of Neurology, Kaohsiung Medical University Hospital, Kaohsiung, Taiwan; ^3^ Neuroscience Research Center, Kaohsiung Medical University, Kaohsiung, Taiwan; ^4^ Department of Neurology, Kaohsiung Municipal Ta-Tung Hospital, Kaohsiung, Taiwan; ^5^ School of Post-Baccalaureate Medicine, College of Medicine, Kaohsiung Medical University, Kaohsiung, Taiwan; ^6^ Department of Pharmacy, Taichung Veterans General Hospital, Taichung, Taiwan; ^7^ Intelligent System Laboratory, Department of Electrical Engineering, College of Electrical Engineering and Computer Science, National Kaohsiung University of Science and Technology, Kaohsiung, Taiwan; ^8^ National Institute of Cancer Research, National Health Research Institutes, Tainan, Taiwan; ^9^ Center for Big Data Research, Kaohsiung Medical University, Kaohsiung, Taiwan; ^10^ Dementia Center, Department of Neurology, Linkou Chang Gung Memorial Hospital, Taoyuan, Taiwan; ^11^ School of Medicine, College of Medicine, Chang Gung University, Taoyuan, Taiwan

**Keywords:** dementia, information technology, mobile applications, case management, behavioral and psychological symptoms of dementia (BPSD)

## Abstract

**Background and objectives:**

This study aimed to confirm the effectiveness of the virtual passport for dementia care.

**Research design and methods:**

The virtual passport is an application (app) tool connecting healthcare professionals, dementia care sites, and people living with dementia (PLWD) and their family caregivers. This tool assists case managers in hospitals by providing individualized care plans and health education to PLWD and their caregivers. The dementia quality indicator achievement rates, care needs investigation and fulfillment, severity of behavioral and psychological symptoms of dementia (BPSD), and changes in caregiver burden and depression are measured at the initial interview and 6 and 12 months after the intervention.

**Results:**

We enrolled 57 and 54 patients and their caregivers in the virtual passport and routine care groups, respectively. Compared to the control group, six quality indicators in the passport group showed significantly higher achievement at 6 months after using the virtual passport. Case managers addressed more care needs at 6 months (1.37 vs 0, p < 0.001) and 12 months (1.32 vs 0, p < 0.001). Improvement in severity of neuropsychiatric symptoms (neuropsychiatric inventory (NPI) irritability/lability difference: -0.58 vs 0.22, p = 0.044; NPI agitation/aggression difference =-0.78 vs 0.00, p = 0.042) were also observed. No obvious influence was found in caregiver burden and depression after using the virtual passport.

**Discussion and implications:**

The virtual passport is an effective information technology tool in improving the quality of dementia care, assisting case management in identifying more care needs, and reducing the severity of BPSD.

## Introduction

Dementia is a degenerative disease characterized by a general decline in cognitive abilities, impacting memory, judgment, language, and behavior. It also often leads to delusions, hallucinations, and disruptive behaviors, imposing a huge burden on both patients and their caregivers (1). As the prevalence and incidence of dementia increase with age, dementia care has become a crucial medical concern in developed countries with a high proportion of elderly populations (2–4).

A population-based study in Taiwan in 2011 reported that the age-adjusted prevalence of dementia among individuals aged 65–69, 70–74, 75–79, 80–84, 85–89, and 90 years was 3.40%, 3.46%, 7.19%, 13.03%, 21.92%, and 36.88%, respectively. The prevalence of dementia exhibited an upward trend with advancing age, doubling approximately every 5-year after age 70 (5). Another study utilizing Taiwan’s National Health Insurance Research Database spanning from 2004 to 2010 indicated a significant increase in the prevalence of dementia and Alzheimer’s disease (AD), rising from 4.7 to 7.6 per hundred people and 2.3 to 3.5 per hundred people over a seven-year period, respectively (6). These findings suggest that dementia care has emerged as a critical concern in Taiwan’s long-term care services owing to its rapidly aging society.

To further support people living with dementia (PLWD) and alleviate the burden of caregivers, the Taiwanese government brought people with dementia aged over 50 years within the Long-Term Care Act 2.0 since 2017. This act introduced two kinds of dementia care services centers: Dementia-integrated Care Centers and Community Service Centers for Dementia (CSCD). Dementia-integrated Care Centers are in hospitals or sites; they assist patients with suspected dementia to obtain accurate diagnoses, provide medical treatment and care management, and introduce social resources. Dementia-integrated Care Centers also provide professional health training and promote public education on dementia literacy. CSCD are community care sites offering cognitive training or stimulation programs, respite services for PLWD, and training programs and support for caregivers (7). As of September 2021, there were 103 Dementia Integrated Care Centers and 506 CSCD in Taiwan, providing care for 55,360 and 16,053 PLWD, respectively (7).

The primary mission of Dementia Integrated Care Centers is to provide case management, which involves a collaborative process of assessment, planning, facilitation, coordination, and evaluation to meet the health needs of individuals and their families. This can help promote the achievement of dementia quality indicators, facilitate the transfer of appropriate social resources, and improve the outcome for both patients and caregivers, such as reducing the severity of neuropsychiatric symptoms (8, 9). However, each care center developed its own content and offered individualized characteristic services and the actual efficacy and chilits of these services for patients and their caregivers are lacking in Taiwan. (10)

The complexity of dementia case management is further compounded by changes in household dynamics. In Taiwan, the nuclear family is the predominant household type and aging parents are often cared for by different children in rotation, and this might require movement between different countries. Thus, PLWD may need to visit various regional hospitals or dementia service stations. Despite the Taiwanese government’s promotion of a national dementia policy, there still remains a lack of exchange of individual medical information and records between different care centers which may increase the social costs and burden.

Currently, Information Technology(IT) (11–13) and mobile applications(apps) (14–16) have been used widely to improve the quality of dementia health care. A variety of innovative products were reported benefit PLWD and their informal caregivers in their daily lives. (12, 15–20) However, apps designed for health professionals or case manager were relatively few and lack relevant research verification or could not fully meet user needs as being a support tool (21).

Consequently, we propose the concept of a virtual passport as available and comprehensive apps for dementia care in Taiwan. The health professionals and case manager have to regularly assess and upload the medical information, including diagnostic evaluations, personalized health education, and care plans developed through shared decision-making with PLWD and caregivers on a secure and encrypted cloud platform. Using a webpage and app, medical information can be shared between different care centers so that the case management could be more consistent and would not be interrupted based on PLWD’s location. Also, family caregivers can access the care plan as well as communicate with healthcare professionals or staff at the CSCD readily.

The objective of this study is to demonstrate the effectiveness of the virtual passport designed to support dementia care. Our aim is to establish an evidence-based integrated information technology (IT) system that connects three stakeholders: healthcare professionals in Dementia Integrated Care Centers, staff in the CSCD, and patients and family caregivers, facilitating effective communication and resulting in a holistic and continuous approach to dementia care.

## Research design and methods

### Design and participants

This case-control study was conducted at the Dementia Center of Changhua Christian Hospital, a medical center in Changhua, Taiwan, from October 2022 to October 2023. The study enrolled patients aged from 50 to 100 who had been diagnosed with mild cognitive impairment or dementia by neurologists or psychiatrists. All participants had a clinical dementia rating (CDR) scale of ≥ 0.5 as evaluated by clinical psychologists (22). The exclusion criteria in this study were patients residing in long-term care facilities or those lacking a family caregiver. Participants were invited to join the virtual passport group if they had access to a computer, mobile phone, or tablet and agreed to have their medical information uploaded to the cloud platform and accessed via the website or mobile app. The control group received routine dementia collaborative care, while the case group utilized the virtual passport in addition to routine dementia care.

This study received approval from the Institutional Review Board of Changhua Christian Hospital (CCH IRB 220128). All participants provided informed consent before participating in the study.

### Intervention method

#### Dementia collaborative care model

Since October 2014, we have a dementia collaborative care model at Changhua Christian Hospital. Our team members include physicians (including neurologists, psychiatrists, gerontologists, and primary care physicians), psychologists, social workers, dieticians, occupational therapists, pharmacists, and nursing case managers. Upon diagnosing mild cognitive impairment or dementia, our care team conducts patient and caregiver interviews to assess various aspects, including the patient’s cognitive, functional, behavioral, and psychological symptoms, as well as the caregiver’s burden, care problems, and preference for utilizing care resources.

The care team addresses 26 different care needs, mostly related to quality measures and the needs of the patients and their caregivers (23–25). Detailed content regarding the assessment for each care need has been published in our previous study (26). When assessments reveal abnormalities, the care team addresses these needs and introduces corresponding education and resources. After the initial interview, the care team arranges monthly phone contacts and face-to-face evaluations every six months to review the care needs of patients and their caregivers. Case managers also examine whether each care need has been fulfilled six months after the need was chosen. For example, if PLWD are still driving or riding a motorcycle, the care need called “driving evaluation and suggestion” will be chosen (26). Any unsafe driving scenario will be discussed with the PLWD and their caregivers. The need will be considered fulfilled if the PLWD can identify more than one risk factor related to unsafe driving/riding and if they can suggest more than one alternative transportation option.

##### Virtual passport for dementia care

“Virtual passport for dementia” is a website/phone app. Its content refers to the 2015 Dementia Management Quality Measurement Set of the American Academy of Neurology Institute (AANI) (24) and the care needs addressed by the memory clinic of Changhua Christian Hospital (26–28). Nine dementia quality indicators are incorporated in the virtual passport (**Table 1**) to regularly assess the care needs of patients and their caregivers.

**Table 1:** Dementia quality indicators in visual passport.

Quality indicators	Brief description	Data upload frequency
Disclosure of diagnosis	**Diagnosis of dementia syndrome**: Explanation of a specific disease identified through prior diagnostic evaluation as the most likely cause of dementia. Ex: Alzheimer’s disease, vascular dementia **General cognitive function assessment**: Clinical dementia rating (CDR), Cognitive abilities screening instrument (CASI), Mini-mental state examination (MMSE) and other cognition assessment scales.	Diagnosis: Evaluated by neurologists and rechecked by healthcare team every 12 months Cognitive scores: Every 12 months
Education and support of caregivers	**Health education resources**: If PLWD has specific symptoms, healthcare team will provide education and suggestions. **Long-term care resources**: Six kinds of long-term care resources currently available in Taiwan are suggested according to patients/caregiver’s need, including assisted devices and home modification, personal and professional care, respite care, transportation services, community aging care centers, and caregiver support centers. Also, family caregiver can use the link to find nearest suitable long-term care center or free caregiver counselling.	Every six months
Functional status assessment	**Assessment of functional status**: Instrumental activities of daily living (IADL), Activities of daily living (ADL).	Every six months
Screening and management of behavioral and psychiatric symptoms	**Assess the severity of BPSD**: Neuropsychiatric inventory (NPI), Cohen-Mansfield agitation inventory (CAMI), Geriatric depression scale (GDS) **Management of BPSD**: Non-pharmacological behavior and lifestyle modifications and pharmacological treatments, including medication’s name, class, does, and side effects	Every 12 months Every six months if having medical treatment
Safety concerns screening and follow-up	**Safety concerns**: Danger to self (patient) or others (caregivers and other individuals), environmental risks, and financial mismanagement **Recommendations if screening were positive**: Providing education and resources	Every six months
Driving screening and follow-up	**Screening for driving risks:** Identifying patients at risk for unsafe driving according to the Taiwanese version of dementia and driving decision aid (DDDA) **Recommendations if screening was positive**: Providing health education and alternatives	Every six months
Advance care planning and palliative care counselling	**Advance care planning (ACP)**: Patients with dementia who received OR declined an advance care plan or surrogate decisions maker documented in the medical record **Palliative care counselling**: Patients with dementia or their surrogate decision maker who received OR declined comprehensive counseling regarding ongoing palliation management and end of life decisions within two years of initial diagnosis.	Every 12 months if declined
Pain assessment and follow-up	**Pain assessment tools**: Visual analogue scale (VAS), Pain assessment in advanced dementia (PAINAD) **Pain reduction intervention**: Health education and pharmacological treatments, including medication name, class, does, and side effect	Every six months Every six months if having pharmacological treatments
Pharmacologic treatment of dementia	**Anti-dementia medications**: Medication name, classes, does, and side effect	Every six months if having pharmacological treatments

The virtual passport group’s medical information and individualized health education are uploaded to a cloud platform. The incorporation of information into the virtual passport enables case managers to address the care needs once again and provide relevant care plans and health education on the passport. Patients and family caregivers can view the evaluation reports, care plans, and health education by logging into the websites or app. Case managers will evaluate and upload information on the virtual passport every six to 12 months (see **Table 1**). Virtual passport for dementia” website/APP was created by Changhua Christian hospital, Kaohsiung Medical University, and National Kaohsiung University of Science and Technology. Android APP V1.0 and website system V1.3.27 were used for the present analysis.

### Measurements

#### Measurement of patient and caregiver features

The sex, age, dementia subtype, baseline clinical dementia rating (CDR) scale, anti-dementia (cholinesterase inhibitors or memantine), perfusion drugs (such as piracetam or ginkgo biloba), psychotropic drugs (antipsychotic, antidepressant, sedative or anti-epileptic) and baseline neuropsychiatric inventory (NPI) scores of patients as well as the baseline Zarit Burden Interview instrument (ZBI) and Center for Epidemiologic Studies Depression Scale (CES-D) scores of the caregivers were collected during the initial assessment. The NPI score was used to assess the severity of dementia-related psychological and behavioral symptoms (29). The total NPI score, which encompasses 12 domains, ranged from 0 to 144, with higher scores indicating greater severity. ZBI was used to evaluate the caregiver burden (30). The total ZBI score ranged from 0 to 88, with higher ZBI scores indicating increased caregiver burden. CES-D was used to assess the caregiver’s depressive mood (31). The total CES-D score ranged from 0 to 60, with higher scores indicating greater caregiver depression.

#### Measurement of virtual passport efficacy

The assessment was conducted at the initial interview and six and 12 months after using the virtual passport. We analyzed the virtual passport efficacy by comparing the following outcome measures between the control and passport groups: (1) achievement rates of dementia quality indicators suggested by the American Academy of Neurology (AAN) 2015 dementia care quality indicators, (2) investigation and fulfillment of care needs, (3) severity of behavioral and psychiatric symptoms, (4) change in caregiver’s burden, and (5) change in caregiver’s depression.

In (2), investigation and fulfillment of care needs, we counted the number of care needs chosen by the case managers and the percentage of need fulfillment six months later. In (3), severity of behavioral and psychiatric symptoms, we analyzed the difference in total NPI scores and each NPI subdomain score between the initial assessment and that six or 12 months later. In (4), change in caregiver burden, we compared the ZBI score at six and 12 months with that at the initial assessment. In (5), change in caregiver depression, we tracked the changes in the CES-D scores.

### Statistical analyses

All data were analyzed using R software (R Foundation for Statistical Computing). Pearson’s chi-squared test or Fisher’s exact test were employed to test for differences in categorical data. Numerical data were tested using Student’s t-test or the Kruskal-Wallis rank sum test. Differences were considered statistically significant when the p-value was less than 0.05.

## Results

This study enrolled 111 patients newly diagnosed with dementia, comprising 54 participants who received routine dementia collaborative care and 57 who used the virtual passport in addition to routine care. **Table 2** presents the baseline characteristics of the participants. There were no significant differences between the control and passport groups with regard to sex, age, diagnosis, CDR scale, medication use and baseline NPI, ZBI and CES-D scores.

**Table 2:** Baseline characteristics of participants.

	Control (N=54)	Passport (N=57)	*p* value
Male	19 (35%)	21 (37%)	0.9
Age	78.9 (6.9)	78.4 (6.5)	0.7
Diagnosis			0.8
AD VaD Other	34 (63%) 8 (15%) 12 (22%)	41 (72%) 5 (8.8%) 11 (19.2%)	
CDR Scale			0.3
0.5 1 2	36 (67%) 11 (20%) 7 (13%)	39 (68%) 15 (26%) 3 (5.3%)	
Pharmacological management			
Antipsychotic Antidepressant Sedative Antiepileptic Dementia drug Perfusion drug	13 (24%) 12 (22%) 12 (22%) 4 (7.4%) 40 (74%) 21 (39%)	10 (18%) 11 (19%) 6 (11%) 5 (8.8%) 41 (72%) 19 (33%)	0.4 0.7 0.095 >0.9 0.8 0.5
Neuropsychiatric inventory (NPI) (pre-test)	5.6 (8.9)	11.5 (20.3)	0.13
Zarit’s caregiver burden interview (ZBI) (pre-test)	26.5 (17.4)	27.7 (16.6)	>0.9
Center for Epidemiological Studies Depression Scale (CES-D) (pre-test)	11.9 (9.7)	12.1 (10.1)	0.9

### Dementia quality indicator achievement rates suggested by AAN 2015 dementia care quality indicators

**Table 3** presents the dementia quality indicator achievement rates after using the virtual passport for six months and 12 months. Compared with the control group, the passport group showed significantly higher achievements six months after virtual passport intervention in six quality indicators, including functional status assessment (85% vs 96%, p=0.049), screening and management of behavioral and psychiatric symptoms (85% vs 96%, p=0.025), safety concerns screening and follow-up (59% vs 100%, p<0.001), driving screening and follow-up (59% vs 98%, p<0.001), advance care planning and palliative care counselling (57% vs 100%, p<0.001), and pain assessment and follow-up (83% vs 98%, p=0.007). Although not significant, all the quality indicators showed higher achievements 12 months after using the virtual passport.

**Table 3:** Dementia quality indicator achievement rates.

Quality indicators	Half-year achievement (N=111)			One-year achievement (N=52)		
	Control (N=54)	Passport (N=57)	*p*	Control (N=25)	Passport (N=27)	*p*
Disclosure of diagnosis	53 (98%)	57 (100%)	0.5	24 (96%)	27 (100%)	0.5
Education and support of caregiver	53 (98%)	57 (100%)	0.5	25 (100%)	27 (100%)	1
Functional status assessment	46 (85%)	55 (96%)	0.049	23 (92%)	27 (100%)	0.2
Screening and management of behavioral and psychiatric symptoms	49 (85%)	55 (96%)	0.025	22 (88%)	27 (100%)	0.10
Safety concerns screening and follow-up	32 (59%)	57 (100%)	<0.001	24 (96%)	27 (100%)	0.5
Driving screening and follow-up	32 (59%)	56 (98%)	<0.001	24 (96%)	27 (100%)	0.5
Advance care planning and palliative care counselling	31 (57%)	57 (100%)	<0.001	24 (96%)	27 (100%)	0.5
Pain assessment and follow-up	44 (83%)	56 (98%)	0.007	23 (92%)	26 (96%)	0.6
Pharmacologic treatment of dementia	53 (98%)	57 (100%)	0.5	25 (100%)	27 (100%)	1

### Investigation and fulfillment of care needs

**Table 4** shows no significant differences in number of baseline care needs between the control and passport group by six (n=2.59 vs 2.11, p=0.053) and 12 (n=1.36 vs 1.41, p=0.7) months of intervention. However, the case manager addresses significantly more care needs after six (1.37 vs 0, p<0.001) and 12 (1.32 vs 0, p<0.001) months of virtual passport intervention. There were no significant differences in care need fulfillment percentage between the control and passport groups by six and 12 months.

**Table 4:** Investigation and fulfillment of care needs.

	Use half year (N=111)			Use one year (N=52)		
	Control (N=54)	Passport (N=57)	*p*	Control (N=25)	Passport (N=27)	*p*
Baseline care needs chosen by case manager	2.59 (1.55)	2.11 (1.48)	0.053	1.36 (0.76)	1.41 (0.75)	0.7
Additional care needs selected after using passport	0	1.37 (1.30)	<0.001	0	1.32 (0.57)	<0.001
Numbers of care need considered fulfilled	N=43 2.30 (1.23)	N=51 2.06 (1.43)	0.053	N=23 1.17 (0.78)	N=26 0.96 (0.93)	0.20

### Severity of behavioral and psychiatric symptoms

**Table 5** shows the change in NPI scores from the initial assessment six and 12 months after passport use. In all, 104 participants completed six months of intervention by the care team. In a comparison with the control group (n = 49), PLWD in the passport group (n = 55) showed significant improvement in irritability/liability (NPI difference =-0.58 vs 0.22, *p*=0.044) after the intervention. Furthermore, 52 participants completed 12 months of intervention. In a comparison with the control group (n = 25), PLWD in the passport group (n = 27) showed significant improvement in agitation/aggression after the intervention (NPI difference =-0.78 vs 0.00, *p*=0.042).

**Table 5:** Severity of behavioral and psychiatric symptoms.

NPI score difference	Use half year			Use one year		
	Control (N=49)	Passport (N=55)	*p*	Control (N=25)	Passport (N=27)	*p*
Total mean (SD)	-0.98(6.36)	-5.96(20.8)	0.079	-0.80(10.1)	-4.33(10.9)	0.2
Delusion	0.08(1.66)	0.11(2.27)	0.9	0.12(2.09)	-0.07(1.82)	>0.9
Hallucination	-0.10(0.87)	-0.09(1.82)	0.4	-0.16(2.23)	-0.52(1.74)	0.2
Agitation	-0.08(1.19)	-0.35(1.39)	0.3	0.00(1.76)	-0.78(2.19)	0.042
Depression	-0.37(1.79)	-0.55(2.36)	0.9	-0.12(2.86)	-0.81(1.98)	0.3
Anxiety	-0.16(1.55)	-0.47(2.60)	0.7	-0.64(1.78)	-0.59(1.89)	0.7
Euphoria	0.00(0.00)	-0.07(0.42)	0.2	0.04(0.20)	-0.04(0.19)	0.2
Apathy	-0.31(2.10)	-0.85(2.96)	0.4	-0.52(3.25)	-0.30(3.11)	0.7
Disinhibition	-0.47(1.66)	0.09(1.54)	0.2	-0.28(1.34)	0.00(0.68)	0.9
Irritability	0.22(1.98)	-0.58(2.68)	0.044	0.64(1.70)	-0.89(2.79)	0.3
Aberrant motor behavior	0.18(1.95)	-0.25(2.20)	0.3	0.20(1.73)	-0.26(1.91)	0.7
Sleep disturbance	-0.86(2.57)	-0.40(2.22)	>0.9	-0.16(2.23)	0.63(2.29)	0.4
Appetite change	-0.08(2.01)	-0.69(2.47)	0.12	0.04(2.32)	-0.30(2.02)	0.5

### Change in caregiver burden

**Table 6** shows the change in ZBI scores from the initial assessment six and 12 months after passport use. In all, 96 (86.5%) and 47 (90.4%) caregivers completed ZBI in six and 12 months, respectively. In a comparison with the passport group (n = 26), the control group (n = 21) showed significant improvement in ZBI scores after 12 months of passport use (ZBI difference =-5.57 vs -0.31, *p*=0.043).

**Table 6:** Change in caregiver burden.

	Use half year (N=111, 86.5% complete)			Use one year (N=52, 90.4% complete)		
	Control (N=45)	Passport (N=51)	*p*	Control (N=21)	Passport (N=26)	*p*
ZBI score (pre-test)	26.5 (17.4)	27.7 (16.6)	>0.9	30.3 (16.9)	28.7 (16.2)	0.5
ZBI score (post-test)	27.7 (14.5)	28.4 (15.5)	>0.9	24.7 (16.9)	28.4 (13.6)	0.3
ZBI score difference	1.18 (15.9)	0.78 (15.2)	>0.9	-5.57 (12.7)	-0.31 (15.5)	0.043

### Change in caregiver depression

**Table 7** shows the change in CES-D scores from the initial assessment six and 12 months after passport use. In all, 96 (86.5%) and 47 (90.4%) caregivers completed ZBI in six and 12 months, respectively. No significant difference was found in CES-D scores between the passport and control groups at both time points.

**Table 7:** The change in caregiver depression.

	Use half year (N=111, 86.5% complete)			Use half year (N=52, 90.4% complete)		
	Control (N=45)	Passport (N=51)	*p*	Control (N=21)	Passport (N=26)	*p*
CES-D score (pre-test)	11.9 (9.7)	12.1 (10.1)	0.9	12.6 (9.3)	10.1 (7.1)	0.4
CES-D score (post-test)	11.8 (8.85)	12.9 (11.8)	>0.9	15.6 (10.7)	12.3 (7.89)	0.5
CES-D score difference	-0.24 (9.92)	0.75 (6.64)	0.6	2.95 (9.81)	2.23 (8.71)	>0.9

## Discussion

This study found that the virtual passport improves the achievement rates of dementia quality indicators, especially for patients newly diagnosed with dementia within six months. Moreover, the virtual passport can help case managers address additional care needs and alleviate the severity of BPSD, particularly irritability and agitation. However, use of the virtual passport showed no obvious influence on the caregiver’s burden or depression.

Case management in dementia care presents significant challenges due to the variability in clinical symptoms, functional status, and home environment of the individual patients. Identifying potential care needs for each case and providing appropriate health education requires substantial effort and time from case managers. In the field of IT use for dementia care, most studies primarily focused on supporting both PLWD and their informal caregivers in their daily lives. A systematic review indicated that most apps developed for PLWD as end users primarily featured reminders/prompts, safety devices and reminiscence/entertainment (16, 32). Another review of dementia-related apps in Australia found the majority of them designed for caregivers with focus on dementia information, practical caregiving, and communication tips (15). However, few studies have considered IT system support case management.

Our earlier study found that a computer-assisted assessment system for dementia case management could improve the quality indicator completion rates for dementia (33). A cluster randomized trial showed that the IT tool may support case managers who are new on the job and emphasize the importance of user-friendliness (34). Moreover, a qualitative study by Thoma-Lürken et al. (21) suggested that a decision support app could aid case managers in clinical judgment, problem assessment, and providing advice on possible solutions. The present study further confirmed the effectiveness of the virtual passport in assisting case managers to identify more care needs and efficiently achieve dementia quality indicators.

Currently, most dementia-related apps designed for PLWD with BPSD focus on non-pharmacological interventions (12, 18, 35). For example, they include music therapy (36), reminiscence therapy (37, 38), and brain games. An open-label study demonstrated that tablet-based mHealth apps can serve as a non-pharmacological management tool for agitation of PLWD with varying severity (39). Some apps have been developed for caregivers or case managers as users, but with little or no significant effect noted in improving BPSD. For example, these apps can advise caregivers on monitoring BPSD severity (40), delivers psychoeducation and caregiver support with the focus on management of BPSD (20) and assist physicians in monitoring the appropriateness of psychotropic drug prescriptions (41).

The present study has confirmed the effectiveness of the virtual passport in reducing the severity of BPSD, particularly in addressing irritability and agitation. The most likely explanation for this is that the dementia collaborative care model was found beneficial for BPSD management in our previous study (9). The virtual passport facilitated close contact between healthcare professionals and caregivers, providing them with more information about non-pharmacological behavior interventions and lifestyle modifications for BPSD.

There is inconsistency on whether mobile applications can improve caregiver fatigue, burden, and depression. Three systematic reviews indicated that technology-based interventions have little or no significant effect on caregiver burden and depression (42–44). These may be attributed to that self-report which is subject to bias from over- and under-reporting of affective responses, a clinical heterogeneity in the interventions and the use of different outcomes made it difficult to compare the effects of different interventions directly. However, some case-control studies have shown that mobile apps can significantly improve family caregivers’ fatigue, burden and depression (17, 19, 20). These apps highlight the importance of caregiver support with focus on aiding management and offering insight into dementia. Also, in a qualitative study, most of enrolled family caregivers prefer easy-to-understand apps and favor concrete intervention strategies (45). Given these findings, our virtual passport can consider providing more health education and practical suggestions for specific symptoms and linking more medical resources with dementia families in the future.

As regards the outcome of the effect on caregiver burden, the control group showed significant improvement in ZBI scores after 12 months of intervention. This observation may be attributed to non-response bias. The response rate in the control group (84%) was lower than that in the passenger group (96.3%), probably because caregivers in the control group experienced increased care burden, leading to withdrawal or loss of follow-up.

The strength of this study lies in its focus on case management. Only a few studies have investigated the effect of IT on case management in dementia care. This study provides this perspective. However, this study has some limitations. First, the study population was relatively small and confined to a single hospital, limiting the generalizability of the findings. Second, the participants in the virtual passport group were invited rather than randomly assigned, leading to the possibility of subject selection bias. Fortunately, the baseline characteristics of the participants showed no significant differences between the control and passport group.

In clinical practice, more evidence-based research studies should be conducted to prove the effectiveness of the virtual passport on the ability to achieve better quality of case management, improve outcomes, and lower society costs. The virtual passport currently supports multiple dementia care sites to use simultaneously. We sincerely hope that virtual passport can cooperate with Taiwan’s government long-term care policies, and more dementia care center and more PLWD and their caregivers can participate in and benefited from it. Besides, the experience and feedback from the end users should be adopted for usability and acceptability evaluation. Safety and security for personal medical information is also an important issue we should focus more. (16, 21, 45). Also, we should consider how to deal with the challenge that these IT tool may be too complex and costly to kept updating, maintain and widespread use (13).

## Conclusion

This study demonstrated that the virtual passport for dementia improved the quality of dementia care, assisting case management in identifying more care needs and attenuating the severity of BPSD. The virtual passport supports dementia care center case managers in providing more efficient and qualitative care to PLWD. It could be a suitable IT tool in clinical practice of healthcare systems in Taiwan. Future studies should consider a larger sample in investigating the effectiveness of the virtual passport on dementia care such as changes of cognition, caregiver’s quality of life, and cost-effectiveness.

## Data Availability

The raw data supporting the conclusions of this article will be made available by the authors, without undue reservation.
